# A single-center prospective study evaluating the usefulness of artificial intelligence for the diagnosis of esophageal squamous cell carcinoma in a real-time setting

**DOI:** 10.1186/s12876-023-02788-2

**Published:** 2023-05-25

**Authors:** Yasuhiro Tani, Ryu Ishihara, Takahiro Inoue, Yuki Okubo, Yushi Kawakami, Katsunori Matsueda, Muneaki Miyake, Shunsuke Yoshii, Satoki Shichijo, Takashi Kanesaka, Sachiko Yamamoto, Yoji Takeuchi, Koji Higashino, Noriya Uedo, Tomoki Michida, Yusuke Kato, Tomohiro Tada

**Affiliations:** 1 Department of Gastrointestinal Oncology, Osaka International Cancer Institute, 3-1-69 Otemae, Chuo-ku, Osaka, 541-8567 Japan; 2 AI Medical Service Inc, Tokyo, Japan

**Keywords:** Artificial intelligence, Endoscopy, Esophageal cancer, Squamous cell carcinoma

## Abstract

**Background:**

Several pre-clinical studies have reported the usefulness of artificial intelligence (AI) systems in the diagnosis of esophageal squamous cell carcinoma (ESCC). We conducted this study to evaluate the usefulness of an AI system for real-time diagnosis of ESCC in a clinical setting.

**Methods:**

This study followed a single-center prospective single-arm non-inferiority design. Patients at high risk for ESCC were recruited and real-time diagnosis by the AI system was compared with that of endoscopists for lesions suspected to be ESCC. The primary outcomes were the diagnostic accuracy of the AI system and endoscopists. The secondary outcomes were sensitivity, specificity, positive predictive value (PPV), negative predictive value (NPV), and adverse events.

**Results:**

A total of 237 lesions were evaluated. The accuracy, sensitivity, and specificity of the AI system were 80.6%, 68.2%, and 83.4%, respectively. The accuracy, sensitivity, and specificity of endoscopists were 85.7%, 61.4%, and 91.2%, respectively. The difference between the accuracy of the AI system and that of the endoscopists was − 5.1%, and the lower limit of the 90% confidence interval was less than the non-inferiority margin.

**Conclusions:**

The non-inferiority of the AI system in comparison with endoscopists in the real-time diagnosis of ESCC in a clinical setting was not proven.

**Trial registration:**

Japan Registry of Clinical Trials (jRCTs052200015, 18/05/2020).

## Background

Esophageal cancer is the seventh most common cancer and the sixth most common cause of cancer-related mortality worldwide [1], and squamous cell carcinoma is the predominant type of esophageal cancer in Asia [2]. The prognosis for advanced esophageal squamous cell carcinoma (ESCC) is poor, but a good prognosis can be expected if ESCC is detected at an early stage and treated with endoscopic resection, chemoradiation, and surgical resection [3–6]. Many studies have reported the usefulness of narrow band imaging (NBI) in the diagnosis of ESCC [7–9] and the diagnostic accuracy of magnifying endoscopy (ME) with NBI is comparable to that of diagnosis by biopsy [10]. However, mastering endoscopic diagnosis takes many years of training, and the skills are difficult for general endoscopists to learn because they do not diagnose ESCC frequently. Furthermore, endoscopic diagnosis of ESCC is prone to interobserver differences [11, 12].

Artificial intelligence (AI) systems for medical diagnostic imaging have evolved rapidly in recent years. Many studies report on the usefulness of AI systems for analysis of the gastrointestinal tract, and we have reported several studies demonstrating the usefulness of AI systems in the diagnosis of ESCC [11–16]. However, a limitation of these studies is that they were conducted using still images or video images, and not on patients in real-time settings. We therefore conducted this study to evaluate the usefulness of an AI system for real-time diagnosis of ESCC in a real clinical setting.

## Methods

### Study design and patients

This single-center prospective single-arm non-inferiority study was performed in accordance with the Declaration of Helsinki. Patients at high risk for ESCC were recruited to maximize the number of lesions detected. Because patients with ESCC have been reported to have a high incidence of synchronous or metachronous recurrence [17, 18] and patients with head and neck cancer also often present with synchronous or metachronous ESCC [19], the inclusion criteria were set as: (1) a history of head and neck cancer or ESCC; (2) no prior surgery, chemotherapy, or irradiation for ESCC (including irradiation of cancer in other organs); (3) an age of between 20 and 90 years; and (4) preserved major organ function. The exclusion criteria were as follows: (1) patients with severe esophageal stricture; (2) patients continuing antithrombotic drugs, for whom biopsy should not be carried out according to the Japanese guidelines (e.g., patients on warfarin and with a prothrombin time international normalized ratio not confirmed to be in the therapeutic range) [20] [21]; and (3) pregnancy or suspected pregnancy. Written informed consent was obtained from all participants.

### Preparation of the training dataset

This study used a training dataset and an AI system developed in previous studies [12], and the following is a summary of the dataset preparation. A deep learning-based AI system for the diagnosis of superficial ESCCs was developed. Endoscopic still and video images of pathologically proven superficial ESCCs captured at three facilities between December 2005 and June 2019 were collected, as were images of noncancerous lesions and normal esophagi captured at Osaka International Cancer Institute between January 2009 and June 2019. After extracting still images from the video images, all the images were manually marked-up by precisely delineating the boundaries and filling in the areas containing superficial ESCCs or other abnormal lesions. All marked images were checked by a board-certified trainer (R.I.) of the Japan Gastroenterological Endoscopy Society. Finally, the training dataset for the AI system consisted of 25,048 images from 1433 superficial ESCCs and 4746 images from 410 noncancerous lesions and normal esophagi. These images included both non-ME and ME with white-light imaging, and NBI or blue laser imaging.

### Construction of the AI system

The AI system was constructed using the same methods as in a previous study [12], and the following provides a summary of this. A BiT-M (ResNet-101 × 1) model pretrained on the ImageNet-21k dataset was used for the AI system. At the transfer learning phase, the model was trained using a BiT-HyperRule to select the most important hyperparameters for tuning. Stochastic gradient descent was used with an initial learning rate of 0.003 and momentum of 0.9. The model was fine-tuned for 3900 steps with a batch size of 32. The learning rate was decayed by a factor of 10 at 30%, 60%, and 90% of the training steps. The model was trained on the dataset and validated using the PyTorch deep learning framework [22]. A difference from the previous study is that to match the quality of the real-time images with that of the training data, a Gaussian filter with a kernel size of 10 was applied and the images were then resized to 512 × 512 for input into the AI system. The trained neural network generated a probability score between 0 and 1 corresponding to the probability of ESCC. A still image was judged as ESCC when the probability score for ESCC surpassed the threshold value in real time.

### Examination protocol

**Fig. 1:**
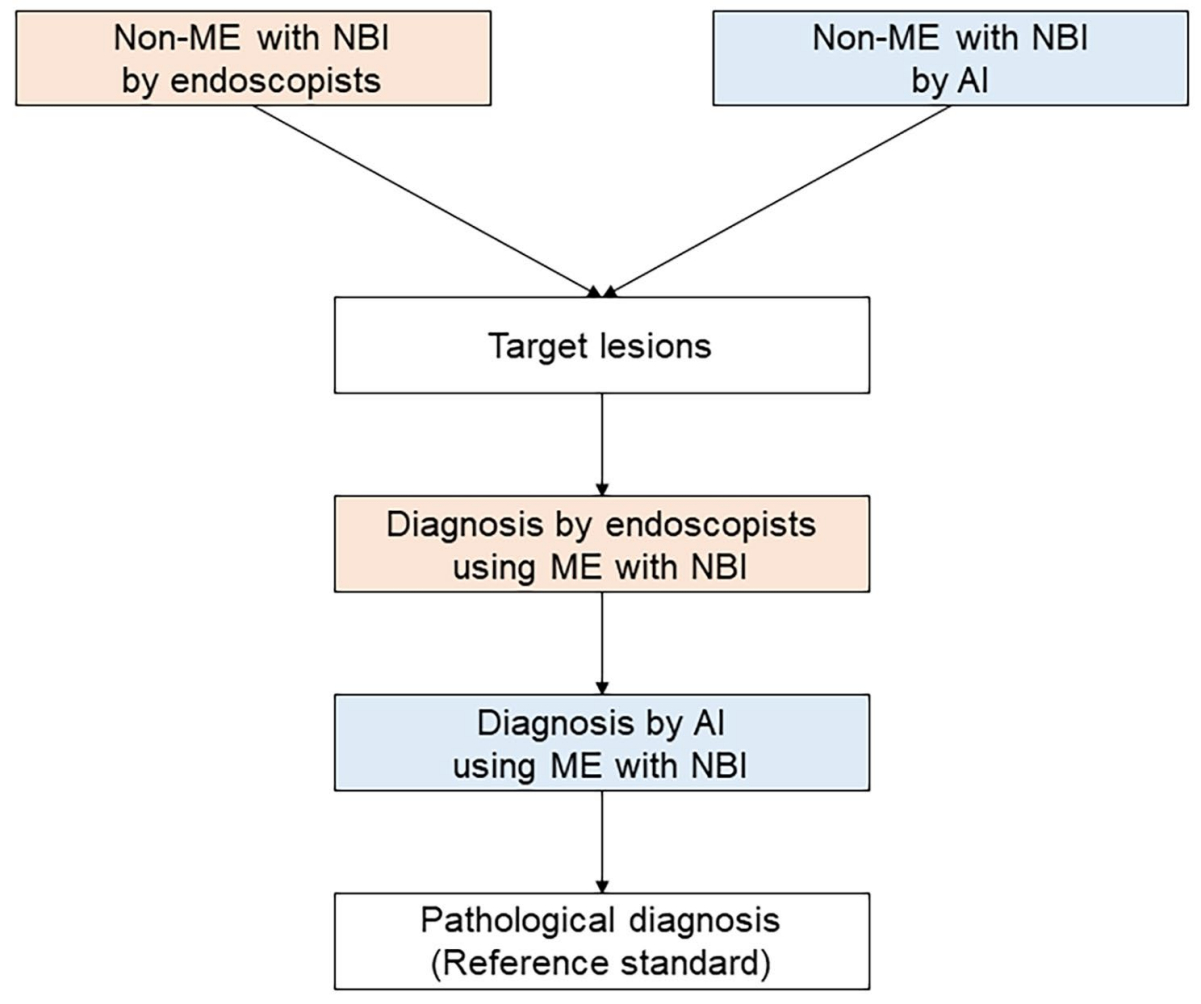
Flowchart of the examination protocol AI, artificial intelligence; ME, magnifying endoscopy; NBI, narrow band imaging

The examination protocol consisted of detection by non-ME with NBI, characterization by ME with NBI, and biopsy of detected lesions (Fig. 1). First, the entire esophagus was observed with non-ME with NBI for at least 10 s, followed by non-ME with NBI using the AI system for at least 10 s to detect target lesions. Target lesions were defined as brownish areas detected by endoscopists that required differential diagnosis between ESCC and noncancerous lesions (e.g., abnormal vessels or esophagitis), or that the AI system judged to be positive for ESCC on non-ME with NBI. Then, the endoscopist classified the target lesions as ESCC or noncancerous lesions using ME with NBI, followed by the AI system differentiating the target lesions between ESCC and noncancerous lesions in real time using captured still images of ME with NBI. All target lesions were ultimately biopsied. Lesions diagnosed as ESCC or suspected to be ESCC were treated by endoscopic resection. The reference standards were histological diagnosis of the resected specimen for resected lesions, and histological diagnosis of the biopsy sample for non-resected lesions. Histological assessments were performed according to the Japanese Classification of Esophageal Cancer [23].

The endoscopic procedures were performed using the following equipment: GIF-H290Z, GIF-H260Z, GIF-Q240Z, GIF-XZ1200, or GIF-EZ1500 endoscopes (Olympus, Tokyo, Japan) with an EVIS X1 system (Olympus, Tokyo, Japan). Five endoscopists who had been routinely diagnosing gastrointestinal tumors performed all endoscopic procedures. These five endoscopists were board-certified fellows of the Japan Gastroenterological Endoscopy Society or had equivalent qualifications, and all had diagnosed over 300 cases of ESCCs with endoscopic images. The five endoscopists had a median endoscopy experience of 8 years (range 6–26 years) and the median number of esophagogastroduodenoscopies performed was 4500 (range 3000–25,000).

### Sample size

A non-inferiority trial design was chosen to investigate the accuracy of the AI system. Our previous study showed diagnostic accuracy of 83% for the AI system when the diagnostic accuracy of endoscopists was 78% ^14^. We considered that we could recommend the use of the AI system for general endoscopists if the lower limit of the 90% confidence interval (CI) for the difference between the accuracy of the AI system and that of endoscopists in a cancer hospital was not less than − 10%. Therefore, we calculated the number of biopsies required with a non-inferiority margin of 10%, a power of 90%, and a one-sided significance level of 0.05, which gave the result of 238 biopsies. According to a previous report [24], 160 target lesions could be expected from 350 participants, and the number of required participants was calculated as 520. Considering a decrease in detection power due to unanalyzable lesions, the planned enrollment of participants was 550.

### Statistical analysis

The primary outcomes were the diagnostic accuracies of the AI system and endoscopists. The secondary outcomes were sensitivity, specificity, positive predictive value (PPV), negative predictive value (NPV), and adverse events. These parameters were calculated as follows: accuracy = correctly diagnosed lesions/total target lesions; sensitivity = correctly diagnosed ESCCs/total ESCCs; specificity = correctly diagnosed noncancerous lesions/total noncancerous lesions; PPV = correctly diagnosed ESCCs/total lesions diagnosed as ESCC; NPV = correctly diagnosed noncancerous lesions/total lesions diagnosed as noncancerous lesions. The results are shown with 95% CIs. All analyses were performed on a personal computer using the EZR software package, version 1.55 (Saitama Medical Center, Jichi Medical University, Tochigi, Japan) [25].

## Results

### Patients and lesions

**Fig. 2:**
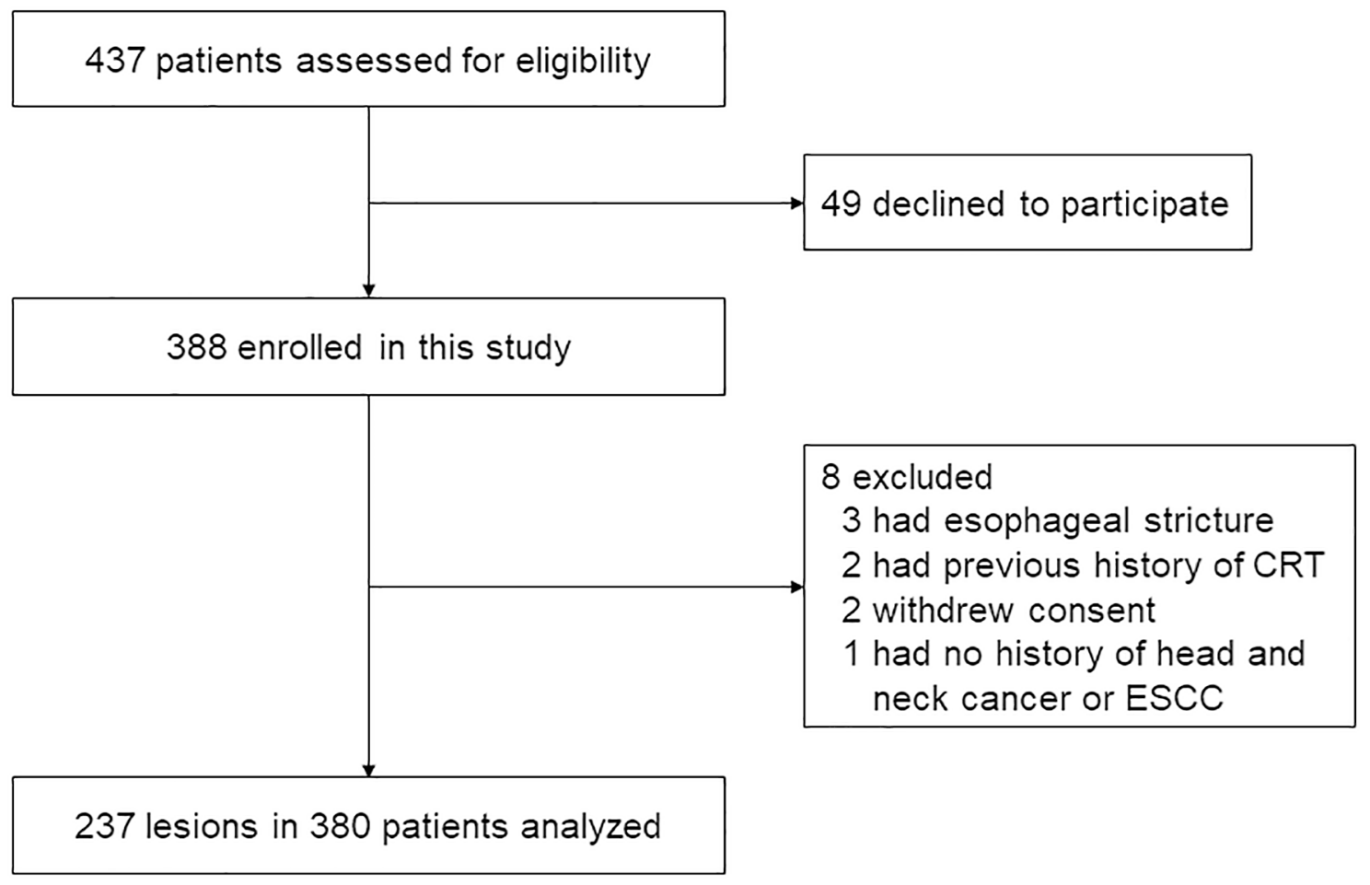
Flowchart of patient inclusion CRT, chemoradiotherapy

Between May 2021 and July 2022, 437 patients were assessed for study eligibility. Forty-nine patients declined to participate, and the remaining 388 patients were enrolled in this study. Patient recruitment was ended after these 388 patients because we had collected 238 biopsied lesions, the calculated sample size requirement for this study. Eight patients were excluded after enrollment: three because of severe esophageal stricture, two because of a previous history of chemoradiotherapy for ESCC, two because of withdrawn consent, and one with no history of head and neck cancer or ESCC. Of the remaining 380 patients, 370 underwent an endoscopic procedure with a GIF-H290Z endoscope, 4 with a GIF-H260Z, 3 with a GIF-Q240Z, 2 with a GIF-XZ1200, and 1 with a GIF-EZ1500. No serious adverse event was observed, and only one patient received intravenous flumazenil because of prolonged deep sedation after the endoscopic procedure. Finally, a total of 237 detected target lesions were evaluated. A flowchart of patient inclusion is shown in Fig. 2.

**Fig. 3:**
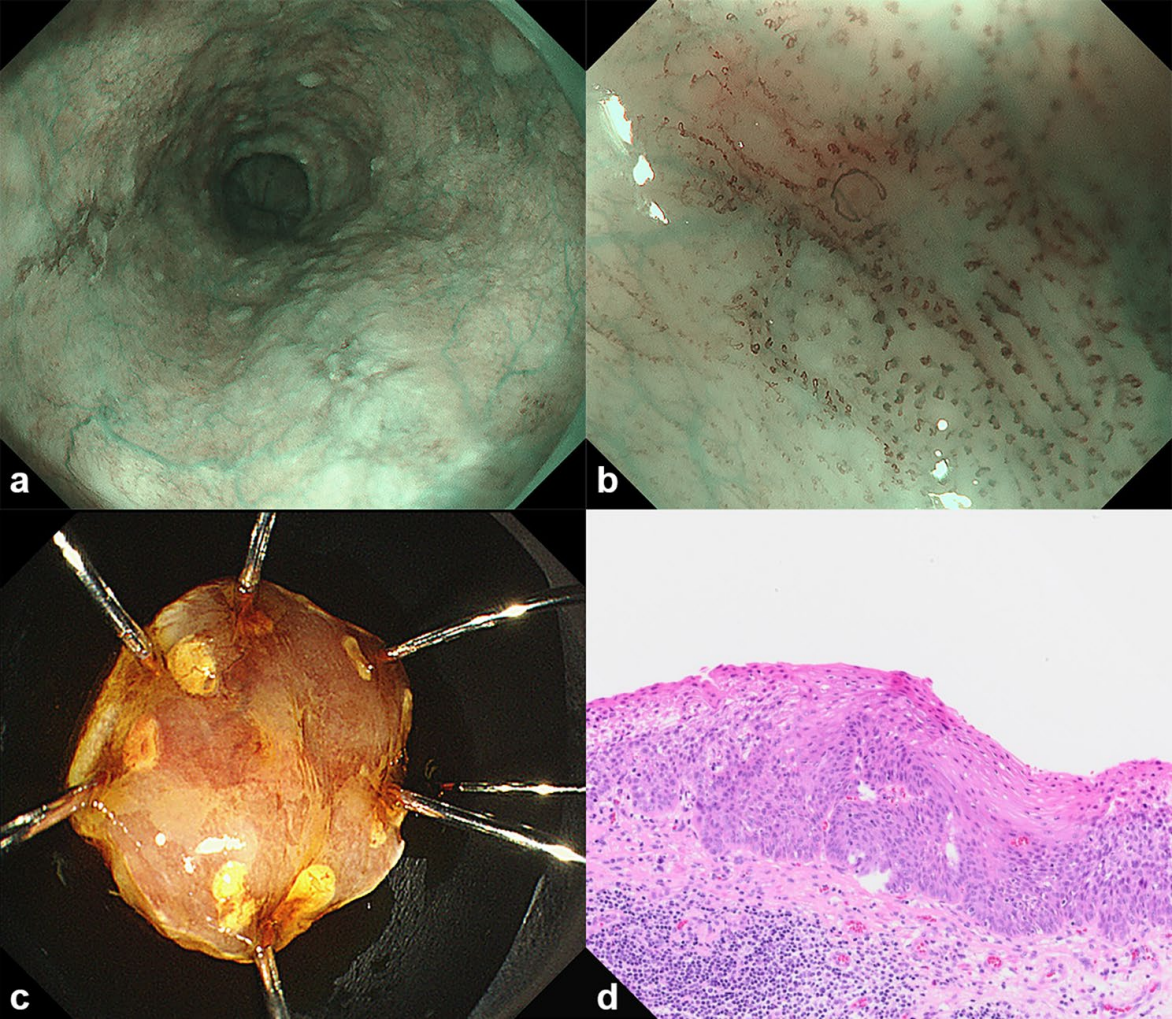
A lesion diagnosed as ESCC by the AI system and an endoscopist a. The lesion was located on the left wall of the lower thoracic esophagus b. Magnifying image of the lesion with NBI. c. Specimen resected by endoscopic submucosal dissection d. The histopathological diagnosis of the resected specimen was squamous cell carcinoma with invasion into the lamina propria AI, artificial intelligence; ESCC, esophageal squamous cell carcinoma; NBI, narrow band imaging

Of the 237 target lesions, 44 were pathologically diagnosed as ESCC. All 44 lesions were superficial. The characteristics of the patients and lesions are shown in Table 1, and a representative case is shown in Fig. 3.

**Table 1:** Characteristics of the patients and lesions

Characteristics			Value
Patient data (n = 380)			
	Sex, male/female		306/74
	Median age, y (range)		72 (49–88)
	Number of lesions, ESCC/noncancerous		44/193
Lesion data (n = 237)			
	ESCC (n = 44)		
		Location: Ce/Ut/Mt/Lt/Ae	1/7/21/14/1
		Median diameter, mm (range)	8 (3–50)
		Macroscopic type: 0-I/0-IIa/0-IIb/0-IIc	0/1/33/10
		Invasion depth^†^: EP/LPM/MM-SM2	17/17/0
	noncancerous (n = 193)		
		SIN/atypical epithelium/esophagitis/no malignancy/others^‡^	36/45/26/75/11

Ae, abdominal esophagus; Ce, cervical esophagus; ESCC, esophageal squamous cell carcinoma; EP, epithelium; LPM, lamina propria; Lt, lower thoracic esophagus; MM, muscularis mucosa; Mt, middle thoracic esophagus; SM, submucosa; SIN, squamous intraepithelial neoplasia; Ut, upper thoracic esophagus.

^†^Ten ESCCs diagnosed by biopsy were excluded from analysis of invasion depth.

^‡^e.g., glycogenic acanthosis and ectopic gastric mucosa

### Performance of the AI system and endoscopists

The AI system diagnosed 30 of 44 ESCCs (68.2%) as ESCC, and 161 of 193 (83.4%) noncancerous lesions as noncancerous lesion (Table 2). In contrast, the endoscopists diagnosed 27 of 44 ESCCs (61.4%) as ESCC, and 176 of 193 (91.2%) noncancerous lesions as noncancerous lesion (Table 2). The accuracies of the AI system and endoscopists were 80.6% and 85.7%, respectively (Table 2). The difference between the accuracy of the AI system and that of the endoscopists was − 5.1% (90% CI, − 10.7–0.6%). The lower limit of the 90% CI was less than − 10%, and we therefore concluded that the non-inferiority of the AI system to endoscopists was not proven.

**Table 2:** Diagnostic performance of the AI system and endoscopists

	AI system [95% CI]	Endoscopists [95% CI]
Accuracy	80.6% (191/237) [75.0-85.4]	85.7% (203/237) [80.5–89.9]
Sensitivity	68.2% (30/44) [52.4–81.4]	61.4% (27/44) [45.5–75.6]
Specificity	83.4% (161/193) [77.7–88.4]	91.2% (176/193) [86.3–94.8]
Positive predictive value	48.4% (30/62) [35.5–61.4]	61.4% (27/44) [45.5–75.6]
Negative predictive value	92.0% (161/175) [86.9–95.6]	91.2% (176/193) [86.3–94.8]

AI, artificial intelligence; CI, confidence interval.

### Subgroup analyses by lesion size and location

Table 3 lists the accuracy, sensitivity, and specificity of the AI system and the endoscopists with respect to lesion size and location. Except for sensitivity for lesions ≥ 10 mm, the AI system had lower accuracy and specificity but higher sensitivity than the endoscopists.

**Table 3:** Subgroup analyses by lesion size and location

	Accuracy		Sensitivity		Specificity	
	AI	Endoscopists	AI	Endoscopists	AI	Endoscopists
Size						
< 10 mm (n = 192)	82.8% [76.7–87.9]	86.5% [80.8–91.0]	61.5% [40.6–79.8]	46.2% [26.6–66.6]	86.1% [79.9–91.0]	91.7% [86.4–95.4]
≥ 10 mm (n = 45)	71.1% [55.7–83.6]	82.2% [67.9–92.0]	77.8% [52.4–93.6]	83.3% [58.6–96.4]	66.7% [46.0-83.5]	81.5% [61.9–93.7]
Location						
Ce-Ut (n = 39)	82.1% [66.5–92.5]	84.6% [69.5–94.1]	100% [51.8–100]	87.5% [47.3–99.7]	77.4% [58.9–90.4]	83.9% [66.3–94.5]
Mt (n = 102)	82.4% [73.6–89.2]	85.3% [76.9–91.5]	61.9% [38.4–81.9]	57.1% [34.0-78.2]	87.7% [78.5–93.9]	92.6% [84.6–97.2]
Lt-Ae (n = 96)	78.1% [68.5–85.9]	86.5% [78.0-92.6]	60.0% [32.3–83.7]	53.3% [26.6–78.7]	81.5% [71.3–89.2]	92.6% [84.6–97.2]

Ae, abdominal esophagus; AI, artificial intelligence; Ce, cervical esophagus; Lt, lower thoracic esophagus; Mt, middle thoracic esophagus; Ut, upper thoracic esophagus

## Discussion

In this study, we evaluated the usefulness of an AI system for real-time diagnosis of ESCC in a clinical setting. However, the non-inferiority of the AI system in comparison with endoscopists in the real-time diagnosis of ESCC was not confirmed.

We attribute the failure to demonstrate the non-inferiority of the AI system to the following reasons. First, the images used for validation of the AI system in the pre-clinical studies [11, 12, 14] were different from those used in the current clinical study. Compressed images were used to train the AI system because the endoscopic images obtained in our routine clinical practice were stored as compressed images. In the validation of the AI system, we used compressed images in the pre-clinical studies, but original uncompressed images in the current clinical study. Although the diagnostic accuracy of the AI system trained on compressed images was high when verified with compressed images in the pre-clinical studies [11, 12, 14], the same high accuracy was not reproduced in the current clinical study using original images. The differences between the original and compressed images are very difficult for humans to discern. However, significant differences do exist between the original and compressed images because we repeatedly confirmed differences in the diagnostic performance of the AI between compressed and uncompressed images from the same dataset. This problem made it difficult to apply our AI system trained on compressed images to original images obtained in the clinical setting. We tried to eliminate this difference between compressed images and original images using various techniques before the start of the study, but were unable to eliminate it completely. We believe that this was the main reason for the negative results of this study. On the basis of the results of this study, we believe that there is a limit to the technical improvements that can be performed to bridge the gap between compressed training images and original images, and that the diagnostic performance of the AI system can be improved in the future by training it with endoscopic images saved in an uncompressed format.

Second, the proportion of ESCC in the target lesions differs from that in previous studies. In previous studies [11, 12, 14], ESCCs accounted for 38.5–56.5% of the validation dataset, whereas in the current study ESCCs accounted for only 18.6% of all target lesions. In the current study, the AI system had high diagnostic accuracy (sensitivity) for ESCC but low diagnostic accuracy (specificity) for noncancerous lesions. The high proportion of noncancerous lesions in this study may have contributed to the lower accuracy of the AI system.

The sensitivity and specificity of the AI system can be altered by adjusting the ESCC threshold, and changing this threshold may improve the overall positive diagnosis rate. However, because sensitivity and specificity have a trade-off relationship, it is not possible to improve both by adjusting this threshold. Because the priority of accuracy, sensitivity, or specificity varies depending on the situation in which an AI system is used in clinical practice, it is necessary to set the optimal threshold based on ROC analysis and the needs of the clinical setting.

In this study, a total of 237 target lesions were detected in 380 participants, which was a higher frequency than expected. We attribute this to the fact that the previous study [24] we used for planning the number of participants was reported more than 10 years ago, and that improvements in endoscopes and endoscopic observation techniques [26] over recent years have increased the number of target lesions detected per participant.

In the subgroup analysis, the sensitivity of the endoscopists was low, especially in lesions < 10 mm. We consider two factors to be the main causes for the low sensitivity of the endoscopists’ diagnoses in our study. One is the prospective study design, which may have resulted in reduced diagnostic performance compared with a retrospective study design [7, 27]. The other reason is the high proportion of cancers that were < 10 mm or that were shallow (cancer invasion depth of epithelium/lamina propria), with the sensitivity for these being generally low [12]. In contrast to the sensitivity, the specificity, which is in a trade-off relationship with sensitivity, was high in this study. Moreover, the diagnostic accuracy for lesions < 10 mm was comparable with that in previous studies [11, 12, 14]. In addition, the difference between the accuracy of the endoscopists and that of the AI system was 3.7% when the lesion size was < 10 mm, but was 11.1% when the lesion size was ≥ 10 mm. In this study, AI diagnosis was made only according to ME findings, while the endoscopists’ diagnoses were also made using non-ME findings as a reference, in addition to ME findings. We speculate that because the significance of non-ME findings is higher in larger lesions, the difference between the endoscopists’ and AI diagnosis was more obvious in lesions ≥ 10 mm than in lesions < 10 mm.

This study is subject to the following limitations. First, this was a single-center study performed at a high-volume center. Second, when the study was planned, we also intended to evaluate the detection and diagnosis of the cancer invasion depth. However, because of the difference between compressed images and original images, estimation of the invasion depth did not improve the accuracy of the AI system, and this feature was not evaluated, thus preventing a comprehensive evaluation of the diagnosis of ESCC. Third, we did not evaluate interobserver agreement or compare diagnostic performance between the endoscopists because the design of this study was not suitable for such assessments. In addition, we have already evaluated these in previous studies [11, 14].

## Conclusions

The non-inferiority of the AI system to endoscopists in the real-time diagnosis of ESCC in a clinical setting was not proven.

## Data Availability

The datasets generated and analyzed during this study are not publicly available but are available from the corresponding author on reasonable request.

## Abbreviations

AI: artificial intelligence
CI: confidence interval
ESCC: esophageal squamous cell carcinoma
ME: magnifying endoscopy
NBI: narrow band imaging
NPV: negative predictive value
PPV: positive predictive value
